# Identification of Characteristic Motor Patterns Preceding Freezing of Gait in Parkinson’s Disease Using Wearable Sensors

**DOI:** 10.3389/fneur.2017.00394

**Published:** 2017-08-14

**Authors:** Luca Palmerini, Laura Rocchi, Sinziana Mazilu, Eran Gazit, Jeffrey M. Hausdorff, Lorenzo Chiari

**Affiliations:** ^1^Department of Electrical, Electronic, and Information Engineering “Guglielmo Marconi”, University of Bologna, Bologna, Italy; ^2^Wearable Computing Laboratory, ETH Zurich, Zurich, Switzerland; ^3^Center for the Study of Movement, Cognition, and Mobility, Neurological Institute, Tel Aviv Sourasky Medical Center, Tel Aviv, Israel; ^4^Department of Physical Therapy, Sackler Faculty of Medicine and Sagol School of Neuroscience, Tel Aviv University, Tel Aviv, Israel; ^5^Health Science and Technologies Interdepartmental Center for Industrial Research (HST-ICIR), University of Bologna, Bologna, Italy

**Keywords:** freezing of gait, wearable sensors, Parkinson’s disease, classification, prediction, inertial measurement unit, machine learning, data analysis

## Abstract

Freezing of gait (FOG) is a disabling symptom that is common among patients with advanced Parkinson’s disease (PD). External cues such as rhythmic auditory stimulation can help PD patients experiencing freezing to resume walking. Wearable systems for automatic freezing detection have been recently developed. However, these systems detect a FOG episode after it has happened. Instead, in this study, a new approach for the prediction of FOG (before it actually happens) is presented. Prediction of FOG might enable preventive cueing, reducing the likelihood that FOG will occur. Moreover, understanding the causes and circumstances of FOG is still an open research problem. Hence, a quantitative characterization of movement patterns just before FOG (the pre-FOG phase) is of great importance. In this study, wearable inertial sensors were used to identify and quantify the characteristics of gait during the pre-FOG phase and compare them with the characteristics of gait that do not precede FOG. The hypothesis of this study is based on the threshold-based model of FOG, which suggests that before FOG occurs, there is a degradation of the gait pattern. Eleven PD subjects were analyzed. Six features extracted from movement signals recorded by inertial sensors showed significant differences between gait and pre-FOG. A classification algorithm was developed in order to test if it is feasible to predict FOG (i.e., detect it before it happens). The aim of the classification procedure was to identify the pre-FOG phase. Results confirm that there is a degradation of gait occurring before freezing. Results also provide preliminary evidence on the feasibility of creating an automatic algorithm to predict FOG. Although some limitations are present, this study shows promising findings for characterizing and identifying pre-FOG patterns, another step toward a better understanding, prediction, and prevention of this disabling symptom.

## Introduction

Freezing of gait (FOG) is a disabling symptom that is common among patients with advanced Parkinson’s disease (PD). FOG is clinically defined as a “brief, episodic absence or marked reduction of forward progression of the feet despite the intention to walk” (1). It most commonly occurs when a person starts to walk, during turning, when passing through narrow passages, and when approaching a destination such as a chair (1). FOG markedly impairs mobility, it is an important cause of falls (2–4) and reduces quality of life (1). External cues such as rhythmic auditory stimulation (e.g., metronome) and visual cues (e.g., walker or stick projecting a laser line on the floor) (5) can help PD patients experiencing freezing to resume walking.

Recently, several research studies used wearable sensors (mostly accelerometer and gyroscopes) in order to quantify the characteristics of FOG events and to implement systems for effective real-time FOG detection. An updated list of these studies can be found in Ref. (6) and in a recent review (4). The most frequent approach for the detection of FOG events is based on the fact that during FOG the acceleration signals recorded by inertial sensors show a pattern of high frequency movements (mostly given by the trembling behavior of the legs) (4, 6–15).

Automatic FOG detection is paramount for providing a cue (such as rhythmic auditory stimulation) during a FOG episode in order to help the person become free of the motor block. FOG prediction on the other hand refers to the ability of predicting FOG before it occurs. By identifying possible precursor signs of FOG (pre-FOG) the cue could be provided as soon as, or ideally just before, the FOG would begin, which might potentially help to prevent the incoming freezing event. Moreover, understanding the causes and circumstances of FOG is still an open research problem (1, 16–25) and so the characterization of the pre-FOG phase, defined as a time window of a few seconds before FOG occurs, might have an extremely relevant impact.

The pre-FOG phase has been studied using different measurement systems, such as camera-based motion capture systems (26, 27), electromyography (28), electroencephalography (29), functional near infrared spectroscopy (16), electrocardiography (17, 30), and skin conductance (30). To the best of our knowledge, only two exploratory studies have used wearable inertial sensors to analyze the pre-FOG phase (31, 32).

In this study, we aimed to use wearable inertial sensors, specifically accelerometers and gyroscopes, to identify and quantify the characteristics of gait during the pre-FOG phase and compare them with the characteristics of gait that do not precede FOG. The hypothesis of this study is based on the threshold-based model of FOG (23) which suggests that before FOG occurs, there is a degradation of the gait pattern. Once the level of deterioration crosses a critical threshold, FOG occurs (23). A classification algorithm was then developed in order to test if it is feasible to predict FOG (i.e., detect it before it happens). The aim of the classification procedure was to identify the pre-FOG phase. An evaluation of the performance of such classifier is presented.

## Materials and Methods

### Overview of Approach

As an initial step toward identification of the pre-FOG phase, we focus here on FOG episodes that take place during movement, excluding FOG episodes that happen after a period of inactivity (i.e., start hesitation). To compare gait and pre-FOG, an *ad hoc* algorithm was designed and implemented to obtain gait and pre-FOG time windows. Then, features which quantify possible patterns leading to FOG were extracted from the identified windows. Finally, a statistical analysis was performed to identify significant differences between gait and pre-FOG. This analysis was performed for each single feature. We also explored the possibility of combining the information from different features by training a classifier to automatically discriminate between gait and pre-FOG.

All the analyses were performed using Matlab (release 2016b, MathWorks, USA).

### Data Set

The CuPiD data set was used for our analyses (30). In this data set, 18 people with PD were monitored during their “ON” medication state using a wearable multisensor setup (30). The study was carried out in accordance with the recommendations of the Ethics Committee of Tel Aviv Sourasky Medical Center with written informed consent from all subjects. All subjects gave written informed consent in accordance with the Declaration of Helsinki. The protocol was approved by the Ethics Committee of Tel Aviv Sourasky Medical Center.

The subjects performed several activities which were selected because they are known to frequently induce FOG (e.g., turning, passing narrow corridors, and dual tasking). The recording protocol included resting periods and other conditions such as completing questionnaires and clinical evaluations [MDS-UPDRS (33), NFOG-Q (34)]. These conditions were not considered in this study as we aimed to analyze only conditions associated with motor activities. The considered conditions are reported in Table 1.

**Table 1:** Protocol conditions.

Condition	Description
Ziegler, Single Task	The Ziegler protocol includes two 360° turns, one 180° turn, and passing through a narrow passage (44). It was performed normally (single task), carrying a glass of water (dual task), and carrying a glass of water while performing serial subtractions (triple task)
Ziegler, Dual Task	
Ziegler, Triple Task	

Figure of 8, Single Task	The subject is required to walk performing a figure of 8 shape five times in a 3-m area. It was performed normally (single task) and with a cognitive dual task, which required to perform serial subtractions or to enumerate words that start with a specific letter
Figure of 8, Dual Task	

Straight + Turns, Single Task	The subject is required to walk straight for 20 m, turn, and walk again on the opposite direction, for five times. It was performed normally (single task), passing a narrow corridor, and with a cognitive dual task, which required to perform serial subtractions or to enumerate words that start with a specific letter
Straight + Turns, Narrow Corridor	
Straight + Turns, Dual Task	

Circles + Random Turns, First Trial	The subject is required to walk in circles, with random 180° and 360° turns, when asked by the clinicians, for a period of 3 min. The condition was repeated a second time for some subjects (second trial)
Circles + Random Turns, Second Trial	

Hospital tour	It includes approximately 10 min of free walking through the crowded hall of the hospital. It includes involuntary stops, turns, changes of direction, using the elevator, and passing through narrow spaces

**Figure 1:**
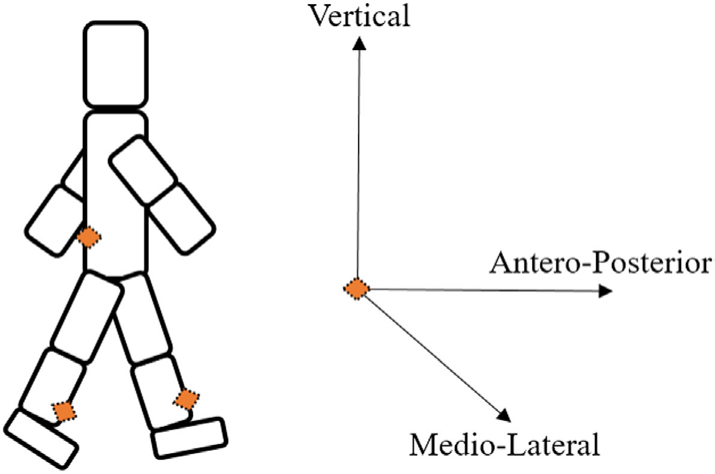
Setup that was considered for the analysis.

In this study, data from 11 subjects were analyzed; only those subjects who exhibited at least one FOG episode during the protocol were included. The subject characteristics are reported in Table 2. We considered the data registered from the two inertial sensors positioned on the shins right above the ankles and from the inertial sensor positioned on the lower back (see Figure 1). The three sensors [ETHOS (35), sampling frequency of 128 Hz] were fixed to the body with straps.

**Table 2:** Subject characteristics.

Subject ID	Age (years)	Disease duration (years)	NFOG-Q	Hoehn and Yahr	MDS-UPDRS Part III
1	89	13	17	4	43
2	55	14	21	3	38
3	63	4	27	4	55
4	68	7	15	3	24
5	63	5	14	3	54
6	60	10	24	3	36
11	64	5	24	2	38
12	77	17	28	4	55
16	81	12	23	3	43
17	49	3	17	2	44
18	76	10	15	3	42
mean	67.7	9.1	20.5	3.1	42.9
SD	11.9	4.6	5.1	0.7	9.3

*NFOG-Q is the new freezing of gait questionnaire (34); MDS-UPDRS Part III is the score of Part III (motor examination) of the MDS-UPDRS (33)*.

### Data Processing

The start and end of each FOG event were identified off-line by two expert clinicians after examining the video recordings [further details in Ref. (30)]. The moment of arrested gait pattern (i.e., stop in alternating left-right stepping) was considered as the start of FOG. The moment when the patient resumed a regular gait pattern was considered as the end of the freezing event.

We defined the period of 2 s before each FOG as the pre-FOG window. We considered 2 s to be the appropriate window length because we were interested in a period of time long enough to capture the last stride before the onset of freezing. In cases where a previous FOG event (or part of it) was present during this 2-s period, the pre-FOG was discarded. This was done because the aim of the work was to study gait before FOG.

To identify gait windows, we first removed the parts of recordings that cannot be considered as gait: pre-FOG windows and FOG events. Then, in the remaining data, we identified continuous portions of at least 2 s. We divided each portion in non-overlapping 2-s windows. In case a portion was not a multiple of 2 s, we discarded an equal period at the beginning and at the end of the portion.

For both gait and pre-FOG windows, we only selected windows with sufficient motion.

**Figure 2:**
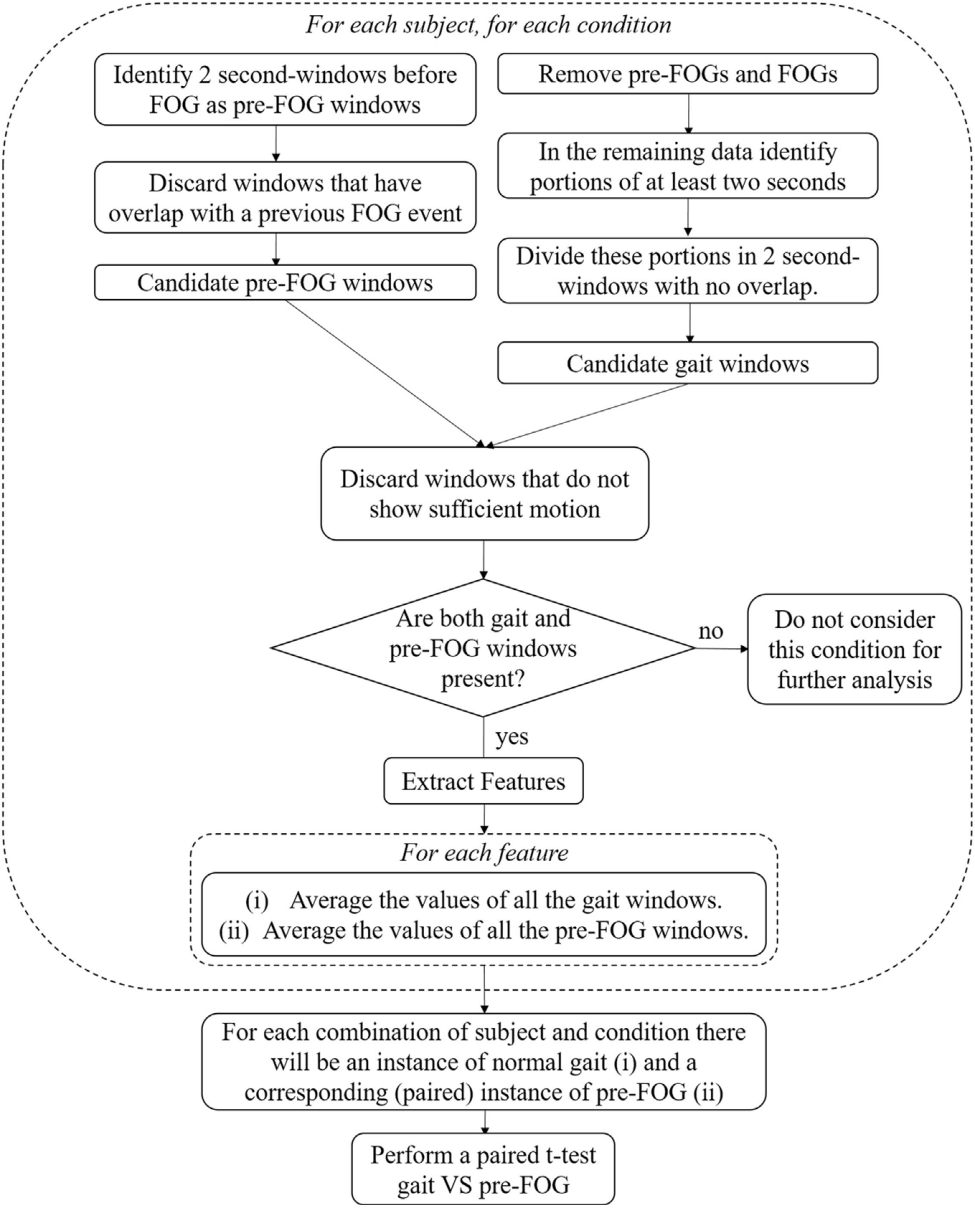
Workflow of data processing.

Consequently, gait windows can be data segments composed of straight walking, curved-path walking (such as the one in the “Figure of 8” and “Circles + Random Turns” conditions), walking through narrow passages, and turns (while walking and in place). The workflow of the identification of gait and pre-FOG windows is presented in Figure 2. The check for sufficient motion in a window is as follows.

A window contains insufficient motion if more than 50% of the samples of that window can be considered motionless.

A sample is considered motionless if two conditions are satisfied simultaneously:

(i) Both left and right ankle norms of the gyroscope signals (angular velocities) are less than 0.5 rad/s.
(ii) The norm of the acceleration of the lower back sensor is in a specific range. To compute this range, first a reference value for the norm of the acceleration was computed by averaging the norm of the acceleration in a portion of the recordings at the beginning of the protocol when the subject was not moving. The range was then defined as a “reference value ± 10% of the reference value.” The reference value is a value near the gravity acceleration *g* = 9.81 m/s^2^ (ideally, it would be exactly *g*).

From each window with sufficient motion, eight features were computed from the signals recorded by the inertial sensors (see Table 3). The features objectively quantify turns (*turning degrees*), gait symmetry (*left-right cross-correlation, left-right difference in SD)*, gait amplitude (*left-right average SD, lower back SD*), and frequency content (*power in the locomotor band, power in the freezing band*, and *freezing index*). These specific features were chosen because we expected them to be sensitive to FOG (1, 23, 36, 37).

**Table 3:** Features extracted from the recorded signals.

Feature	Sensor	Signal	Direction	Description
*Turning degrees*	Lower back	Angular velocity	Vertical	In order to obtain the turning degrees, the angular velocity around the vertical axis was low-pass filtered at 1.5 Hz, then integrated, as in Ref. (45)

*Left–right cross-correlation*	Left and right ankles	Angular velocity	Mediolateral	The cross-correlation between two signals identifies the similarity between them at different lags (shifting in time one signal with respect to the other). This feature is the maximum of cross-correlation between the left and right leg among lags from 0.25 to 1.25 s (this is considered as the period where a pattern of alternate stepping can be in place). If walking is in place there should be a peak of cross-correlation for a lag in that range. As a technical note, the unbiased cross-correlation was performed and the signals were detrended before applying cross-correlation. The angular velocity in the mediolateral direction was chosen because it reflects the leg forward movement during gait for sensors on the ankles (see Figure 3)

*Left–right average SD*	Left and right ankles	Angular velocity	Mediolateral	It is the average between the SD of the signal of the right ankle and the SD of the signal of the left ankle. It is a measure of overall variation and range of leg movement

*Left–right difference in SD*	Left and right ankles	Angular velocity	Mediolateral	It is the absolute difference between the SD of the signal of the right ankle and the SD of the signal of the left ankle. It is a measure of the difference in ranges between the left and right leg

*Lower back SD*	Lower back	Acceleration	Anteroposterior	It is a measure of overall variation and range of motion of the trunk. The anteroposterior direction was chosen to reflect forward motion

*Power in the locomotor band*	Left and right ankles	Acceleration	Anteroposterior	The frequency of walking movements (locomotor activity) is considered to be concentrated around its characteristic periodic patterns, steps, and strides, which are around 2 and 1 Hz, respectively. This feature is the power in the locomotor band, which is defined to be between 0.5 and 3 Hz, as in Ref. (8). This feature was calculated for both left and right ankles and the two values were then averaged

*Power in the freezing band*	Left and right ankles	Acceleration	Anteroposterior	It was found that leg trembling during freezing is characterized by a higher frequency pattern with respect to the one that is characteristic of walking (7, 8, 14, 15). This feature is the power in this freezing band, which is defined to be between 3 and 8 Hz, as in Ref. (8). This feature was calculated for both left and right ankles and the two values were then averaged

*Freezing index*	Left and right ankles	Acceleration	Anteroposterior	It is the ratio between the power in the freezing band and the power in the locomotor band (8). It is usually used in studies for detecting freezing of gait with inertial sensors. When freezing is already in place, this index tends to show a high value (8, 14, 15). This feature was calculated for both left and right ankles and the two values were then averaged

**Figure 3:**
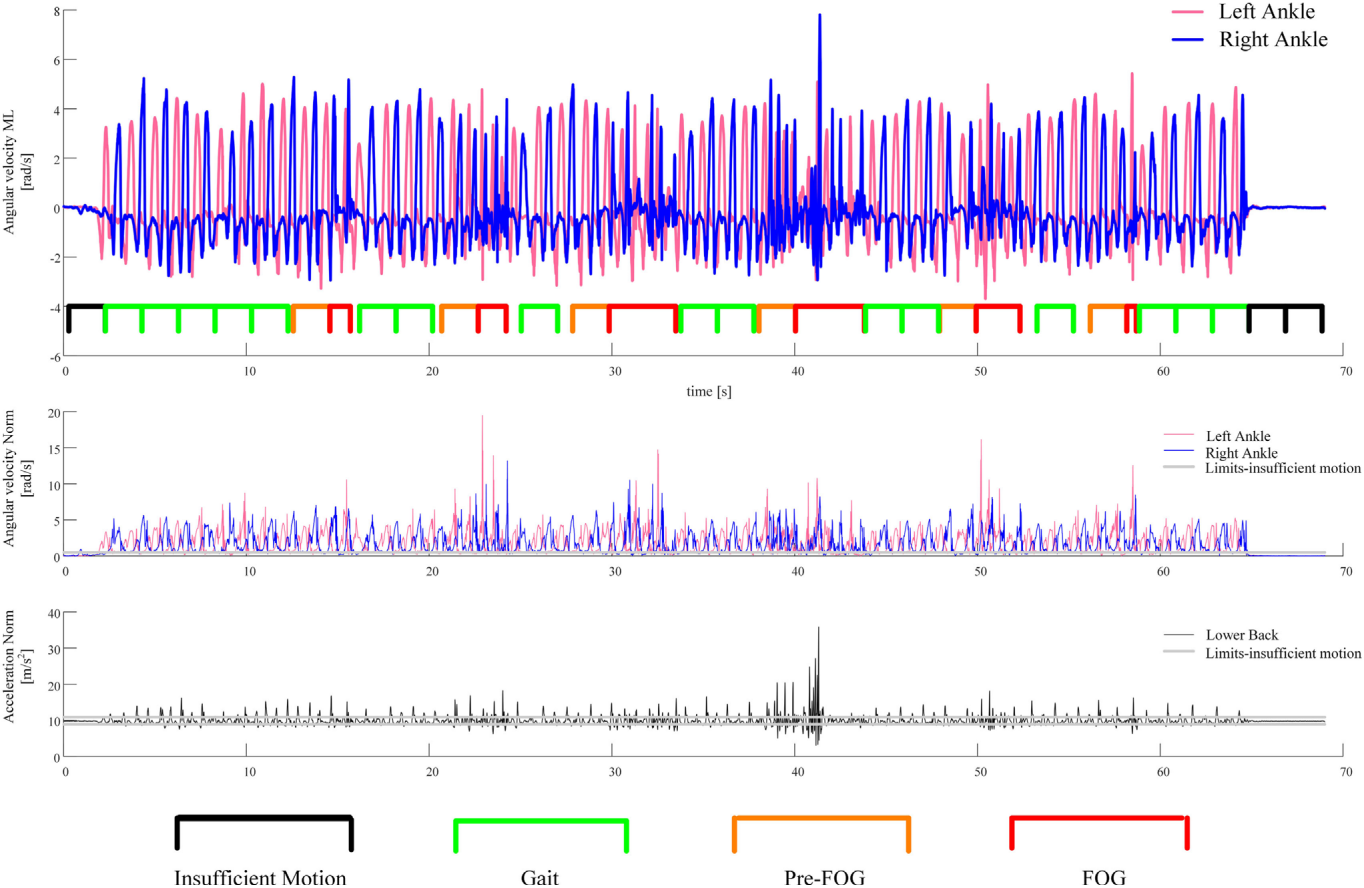
An example of the segmentation of the recorded signal in gait and pre-FOG windows. The first plot from the top shows the recorded angular velocities of the sensors on the left and right ankles together with the segmentation of the windows. The second plot is the norm of the angular velocity of the sensors on the left and right ankles. The third plot is the norm of the acceleration of the sensor on the lower back. These two norms are used to perform the check for sufficient motion in a window.

The result of this process on a representative example is reported in Figure 3, which includes 16 gait windows, six pre-FOG windows, and three windows (which were candidate gait windows) that were discarded because of insufficient motion. The features extracted from the signals of the 16 gait windows and six pre-FOG windows were then averaged, obtaining a single feature value for gait and a corresponding (paired) single value for pre-FOG, respectively. These two paired values were then considered in the statistical tests.

This process was repeated for each subject, for each condition. Only conditions with both gait and pre-FOG windows were selected for further analysis (see Figure 2 and Table 4).

**Table 4:** The conditions that were performed by the subjects are highlighted in green.

Condition/subject ID	1	2	3	4	5	6	11	12	16	17	18
Ziegler, Single Task	x	x	x			x			x		x
Ziegler, Dual Task	x					x			x	x	x
Ziegler, Triple Task		x			x	x	x				x
Figure of 8, Single Task	x	x	x		x	x				x	
Figure of 8, Dual Task	x					x				x	
Straight + Turns, Single Task	x		x			x		xx	x	x	
Straight + Turns, Narrow Corridor	x				x	x				x	
Straight + Turns,Dual Task						x					
Circles + Random Turns, First Trial	x	x			x	x		x	x	x	x
Circles + Random Turns, Second Trial									x	x	
Hospital tour				x					x	x	

All the reported conditions were performed a single time with the exception of subject 12 who performed twice the “Straight + Turns, Single Task” condition. The conditions that were selected for the analysis are the ones with at least one gait window and at least one pre-FOG window. They are marked with an “x.”

### Statistical Analysis

We considered all the pairs (gait–pre-FOG) obtained from the procedure described above and shown in Figure 2. Each pair corresponds to a specific condition of a specific subject. In the pair, the first sample is the average of the feature values of the gait windows, and the second sample is the average of the feature values of the pre-FOG windows in that condition. The paired samples were considered condition by condition to find significant differences that did not depend on the degree of difficulty of a condition. The average was performed to have, for each condition, a single value for gait and a single value for pre-FOG. The averaging allowed for dealing with the imbalance between the number of gait windows and pre-FOG windows. In fact, usually there were more gait windows than pre-FOG windows (see Figure 3). Furthermore, both the number of gait windows and pre-FOG windows changed when considering different subjects and conditions.

We used the paired *t*-test to perform the comparison between gait and pre-FOG. The level of significance *p* was set at *p* = 0.05. Since we performed eight testing procedures (i.e., one for each feature), the results were considered significant if they remained significant after the correction for multiple testing procedures of Benjamini and Yekutieli (38).

### Classification

In order to develop the classifier, we selected the three best features in characterizing the differences between gait and pre-FOG from the statistical analysis (the features associated with the lowest *p*-values). Then, we trained a linear discriminant analysis classifier (39). The classifier was trained to discriminate between two classes: gait and pre-FOG. The trained classifier provided the probability that a certain window was gait or pre-FOG. In order to obtain a fair estimation of the accuracy of the classifier, a leave-one-subject-out procedure was performed: when the classifier was tested on a certain subject, all the data from the remaining subjects were used to train the classifier.

The classifier was trained and tested on all of the gait and pre-FOG windows of the conditions selected for the statistical analysis (i.e., the conditions with both gait and pre-FOG windows).

The performance of the classifier, considering the obtained probabilities, is quantified by the area under the curve and the optimal combination of sensitivity and specificity. The latter is calculated with Youden’s index (40). The threshold on the probability corresponding to this optimal combination of sensitivity and specificity is also provided in Table 5. This threshold is used to classify a window in one of the two classes. So, if the probability of being pre-FOG is higher than the threshold, the window can be classified as pre-FOG (i.e., the classifier predicts an incoming FOG), otherwise the window can be classified as gait.

**Table 5:** Performance of the classifier.

Subject ID	1	2	3	4	5	6	11	12	16	17	18	Mean
Area under the curve	0.69	0.66	0.90	0.79	0.69	0.87	0.90	0.75	0.80	0.51	0.75	0.76
Sensitivity	0.56	0.90	0.92	1.00	1.00	0.88	1.00	0.64	0.74	0.73	0.80	0.83
Specificity	0.79	0.41	0.76	0.79	0.41	0.75	0.67	0.83	0.81	0.37	0.75	0.67
Threshold	0.53	0.26	0.22	0.20	0.23	0.49	0.72	0.19	0.59	0.23	0.57	0.38

## Results

There was a significant amount of variability with respect to performed conditions in the CuPiD data set (Table 4). Not every subject performed the same number of conditions: only one subject (ID 18) performed every condition. The fact that different subjects performed a different number of conditions was mainly due to their health status and disease stage. The clinician decided how many conditions a specific person could perform. For example, the subject who performed the fewest number of conditions (subject 12) was the one with longest PD duration and highest NFOG-Q and MDS-UPDRS Part III scores. The “Straight + Turns, Single Task” condition was the only condition performed by every subject. This condition was likely the easiest to perform (it consists only in straight walking and predefined turns). There was additional intersubject variability with respect to the conditions that had both gait and pre-FOG (i.e., the conditions selected for the analysis). This was due to the episodic nature of FOG as well as due to the fact that different subjects, depending on their disease stage and health status, experienced a different number of FOG episodes.

**Figure 4:**
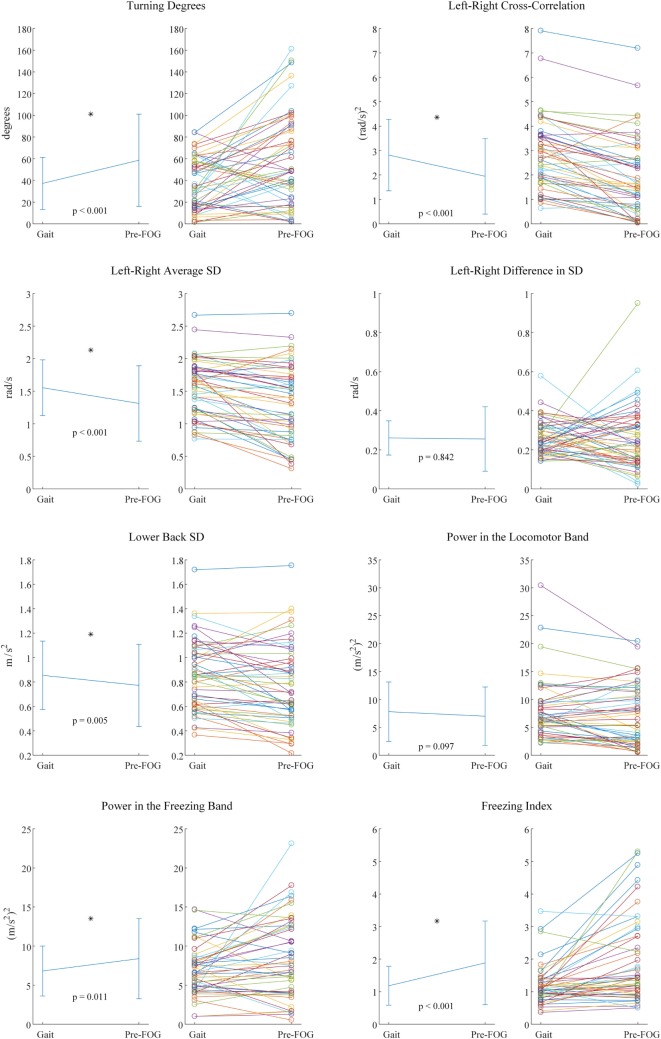
Paired *t*-test results for each feature. For each feature, two plots are present. On the left the mean and SD values are reported, together with corresponding *p*-value and statistical significance (*). On the right, the values of each pair that was considered in the statistical testing are reported.

Fifty paired samples were obtained using the procedure summarized in Figure 2 (the number of paired samples corresponds to the total number of x marks in Table 4). In Figure 4, the *p*-values and the significant features (corrected for multiple testing) obtained from the paired *t*-tests are reported. Six out of eight features showed significant differences between gait and pre-FOG. These features were: *turning degrees, left-right cross-correlation, left-right average SD, lower back SD, power in the freezing band*, and *freezing index*.

**Figure 5:**
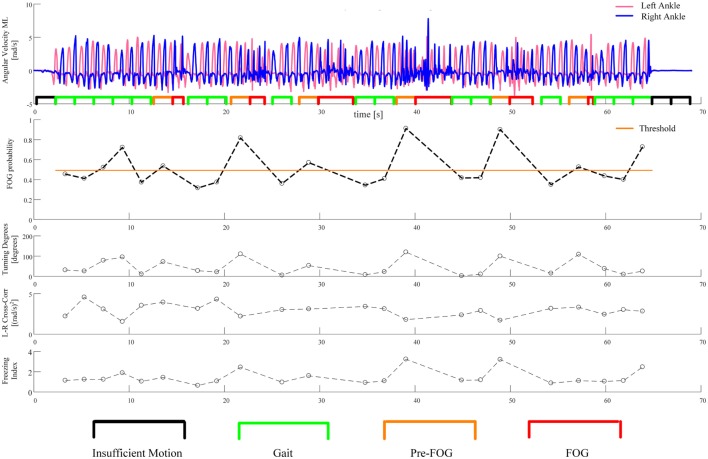
An example of the application of the classifier. The first plot from the top shows the recorded angular velocities of the left and right ankles together with the segmentation of the windows, as in Figure 3. The second plot reports the probability of incoming FOG, as predicted by the classifier. This probability is computed for each gait and pre-FOG window. The threshold on probability is also reported: if the probability is higher than the threshold, then the classifier predicts a FOG (i.e., it identifies the window as pre-FOG), otherwise, the classifier identifies the window as gait. In the last three plots, the values of the three features which are used in the classifier are reported.

The three features with the lowest *p*-values overall were *left–right cross-correlation, left–right average SD*, and *freezing index*. However, the three features selected for the classifier were *left–right cross-correlation, turning degrees*, and *freezing index*. *Turning degrees* (the fourth lowest *p*-value) was selected instead of *left–right average SD* because the latter showed high correlation with *left–right cross-correlation* (*r* = 0.95 considering gait windows, *r* = 0.94 considering pre-FOG windows). An example of the application of the classifier is reported in Figure 5. The performance of the classifier on each subject is reported in Table 5.

## Discussion

Using inertial sensors, we found evidence that the pre-FOG phase differs from gait in patients with PD who suffer from FOG. This finding is consistent with the threshold model of FOG (23) as it suggests that an abnormal movement occurs before the FOG event.

As expected, the higher *turning degrees* registered in the pre-FOG phase reflect the fact that turning triggers FOG. Plotnik et al. (41) showed that subjects who experience FOG have a poor bilateral coordination of stepping. It was hypothesized that tasks requiring a high degree of left–right coordination (such as turning) could predispose to FOG events (41).

We found the *left–right cross-correlation* to significantly decrease during pre-FOG. This feature quantifies both the temporal symmetry between the two limbs and the movement amplitude of both limbs. Therefore, this result shows a reduction of symmetry between the two limbs and of the overall amplitude (range) of movement. The former is consistent with Plotnik et al. (37), where a reduction in symmetry was found in the gait of subjects who suffered from FOG. Although in Nieuwboer et al. (36) this reduction was not found during pre-FOG. The latter is confirmed by the significant reduction that was found in the forward range of leg movements (*left–right average SD*) and of the trunk (*lower back SD*). These results are consistent with that of Ref. (36), where a pattern of reduced movement amplitude before FOG was reported.

The *left–right difference in SD* was not significant. We decided to quantify a possible difference (asymmetry) in movement amplitude (range) between the left and right leg just before FOG; however, this particular difference was not observed in this study.

In the frequency domain, we sought to test the features that are usually used for FOG detection (i.e., after the FOG episode has begun) to see if there was a similar characteristic pattern in the pre-FOG phase. The *power in the freezing band* and the *freezing index* showed a significant difference between gait and pre-FOG, with higher values associated with the pre-FOG phase. This reflects a pattern of high frequency movements that is not only present when FOG is in place (4, 6–13) but also just before it starts. This is in line with the results by Ferster et al. (32), obtained on a subset of the subjects of this study.

The sensing modality that is more similar to the one used in this study is the camera-based motion capture system. There is a trade-off between the advantages and limitations of the two approaches. On one hand, with motion capture, it is possible to have more detailed information about the movement since it is possible to accurately quantify displacement features (e.g., step and stride length) (26), which are not yet reliably quantifiable with inertial sensors, especially when considering pathological gait such as that observed in people with advanced PD. The important limitation of such system, however, is the constrained laboratory environment and the limited working volume. On the other hand, wearable inertial sensors provide the possibility of quantifying relevant features in diverse environments, shifting from laboratory to unconstrained environments, and real-life conditions. For example, Weiss et al. (42) showed that subjects with PD suffering from FOG, who were recorded continuously for 3 days during daily community living, have altered gait variability and consistency with respect to subjects not suffering from FOG. In this study, wearable sensors provided the possibility to test subjects in a series of diverse conditions, the hospital tour being the one most similar to daily living activities. Difference between laboratory and unconstrained activity monitoring is particularly important in FOG, where it is common to find subjects who are reporting freezing events at home, but do not experience FOG in the laboratory (43).

In the previous work by Nieuwboer et al. (26) that used motion capture and found spatiotemporal abnormalities of gait, only FOG events without direction change were selected for the analysis. In contrast, all FOG events, except for those occurring at gait initiation [which were not considered in Ref. (26) either], were considered in this study. We did this because we were interested in quantifying the effect of turning as a trigger of FOG and turning is one of the main activities performed at the time of FOG onset (25). Another difference, which is a limitation of this study, is that subjects of the CuPiD data set did not perform *ad hoc* tasks of voluntary stops. Therefore, we were unable to compare the degradation of gait specific to pre-FOG with gait characteristics prior to voluntary stops.

### Classification

The idea behind the use of the classifier was to replicate the threshold model of FOG (23), by identifying when the combination of values of three gait features changes over a critical threshold. The performance of the classifier varied across subjects. For most subjects, the performance was acceptable, for some subjects (such as the one presented in Figure 5), the performance was good, while for one subject (subject 17), the performance was not better than random classification (Table 5).

The optimal threshold of the classifier reported in Table 5 and Figure 5 can be interpreted as an estimate of the critical threshold of gait degradation of the threshold model of FOG. The optimal threshold varies among subjects, suggesting that different subjects may have different critical levels of gait degradation leading to imminent FOG. This is possibly connected to the different disease stages and functional state. Different optimal thresholds also imply different trade-offs between sensitivity and specificity.

The example in Figure 5 shows that high *turning degrees*, low *left–right cross-correlation*, and a high *freezing index* contribute to an increase in the probability of an incoming FOG episode. In particular, it can be seen that all FOGs are correctly predicted (the first and last one by a small margin). On the other hand, there are three false positives (i.e., windows predicted to be FOG that are actually gait). The first two false positives take place during a turn of approximately 100° (see Figure 5, third plot from the top). The high value of *turning degrees*, together with the reduction in *left–right cross-correlation*, pushes the probability over the threshold. A FOG episode, however, does not occur. The third false positive is at the end of the “Figure of 8,” when the subject stops. Here, the value of *turning degrees* is low and the *left–right cross-correlation* shows an average value. However, the value of *freezing index* is particularly high. This combination of feature values still pushes the probability of incoming FOG over the threshold, but the subject just stops her/his gait (the freezing episode does not occur).

### Limitations and Future Developments

The main limitation of this study is the small number of subjects involved (11 subjects). Another limitation is that subjects with different disease stages performed a different number and type of conditions. In addition, subjects did not experience FOG in every condition that they performed. This was due to both the episodic nature of FOG and the fact that subjects were tested during their “ON” medication (resulting in better than usual gait performance and fewer FOG events).

A consequence of these limitations is that different subjects had a different number of conditions considered in the statistical analysis (Tables 4 and 6). Another consequence is that, for each condition, the number of gait windows that were averaged was higher than the number of pre-FOG windows. In total (considering all subjects, all conditions with both gait and pre-FOG) there were 2,128 gait windows and 137 pre-FOG windows (Table 6). The imbalance between the number of gait and pre-FOG windows may also be attributed to the fact that there were often long periods of straight walking that did not elicit FOG events.

**Table 6:** Detailed number of gait and pre-freezing of gait (FOG) windows for each subject.

Subject ID	Gait windows	Pre-FOG windows	Conditions with both gait and pre-FOG
1	204	16	7
2	111	10	4
3	21	12	3
4	184	1	1
5	162	4	4
6	236	33	9
11	12	4	1
12	275	11	3
16	407	19	6
17	435	22	8
18	81	5	4
Total	2,128	137	50
Mean	193.5	12.5	4.5
SD	139.9	9.5	8.5

In future studies subjects should also be tested during their “OFF” medication state. In this case, we expect to see an increased average FOG probability (i.e., the corresponding dotted line in Figure 5 would shift upwards) because gait performance would on average be worse without medication (23). An increased average FOG probability would then lead to more events over the critical threshold (and, therefore, to more FOG events).

Specific to the classification procedure, the considered windows do not overlap (see Figure 5), so the algorithm was not tested in a situation that fully resembles real-time. Then, in order to consider a real-time application, the processing time of feature extraction and classification on a mobile system should also be evaluated. The performance of the classification algorithm was acceptable for some subjects but not satisfactory for others. In this study, we used a simple classifier in order to find a linear relation between the extracted features. The use of more sophisticated machine learning techniques (which will require larger data sets), together with personalization of the algorithms, will probably yield better performance.

In the study by Mazilu et al. (30), which was performed on the same subjects of this study, it was shown that skin conductance is promising for the identification of pre-FOG phase. Combining this additional data source with movement data could also help to improve the performance in FOG prediction.

We considered a time window of 2 s before FOG as the pre-FOG phase. The previous studies on pre-FOG implemented different window durations. Further studies should analyze what is the most appropriate window duration for analyzing pre-FOG and how (and if) this could change among subjects. It should be further studied whether and to what extent the classifier and the statistical analysis are specific to pre-FOG (e.g., with respect to voluntary stops). For future studies, we would suggest to: (a) select subjects with similar disease stages (possibly stratifying by clinical FOG severity); (b) select a limited number and type of conditions; and (c) include a condition with voluntary stops. This may help to more fully match real-world conditions.

## Conclusion

This work leverages wearable inertial sensors to study the patterns leading to FOG (i.e., the pre-FOG phase). The results indicate that there is a degradation of gait that occurs before freezing, suggesting that there are some identifiable and quantifiable precursors to the event itself. Future work should more fully investigate what factors lead to the observed changes in the movement pattern and how the pre-FOG phase evolves into FOG. The work also presents preliminary evidence for the feasibility of automatic FOG prediction using wearable inertial sensors and classification algorithms. Although some limitations are present, this study shows promising results for characterizing and identifying pre-FOG movement patterns, a first step toward a better understanding, prediction, and prevention of this disabling symptom.

## Ethics Statement

This study was carried out in accordance with the recommendations of the Ethics Committee of Tel Aviv Sourasky Medical Center with written informed consent from all subjects. All subjects gave written informed consent in accordance with the Declaration of Helsinki. The protocol was approved by the Ethics Committee of Tel Aviv Sourasky Medical Center.

## Author Contributions

LP, LR, and LC conceived and designed the study. SM and EG carried out data collection and data preprocessing. LP carried out data analysis and statistical analysis. LP wrote the manuscript. JH and LC supervised the study. All the authors revised and approved the final manuscript.

## Conflict of Interest Statement

LP and LC have a significant financial interest in mHealth Technologies, a company that may have a commercial interest in the results of this research. All other authors declare no competing interests. The reviewer, CL, and handling editor declared their shared affiliation and previous collaboration, and the handling editor states that the process nevertheless met the standards of a fair and objective review.
